# Book Reviews

**Published:** 1904-04

**Authors:** 


The American Yearbook of Medicine and Surgery for 1904. A Yearly Digest of Scientific Progress and Authoritative Opinions in all branches of Medicine and Surgery, drawn from journals, monographs, and textbooks of the leading American and foreign authors and investigators. Arranged, with editorial comments, by American specialists, under the editorial charge of George M. Gould, A. M., M. D. Volume I, *General Medicine*. Octavo, 673 pages, illustrated. Volume II, *General Surgery*. Octavo, 680 pages, illustrated. Philadelphia, New York, London: W. B. Saunders & Co. 1904. (Per volume: Cloth, $3.00 net; half morocco, $3.75 net.)

An examination of the contents of this valuable reference book will at once suggest the enormous labor required to prepare it for publication. Only men of experience and judgment in the selection and condensation of material could produce such satisfactory results within the time limit set. The several departments are presided over by just the kind of men needed to accomplish the task, and over all sits in calm, impartial judgment the master spirit of the enterprise.

The same general plan of make-up and style of printing, as has prevailed for the last few years, continues, except that, beginning with the present issue and to be continued in the future, is a general summary of the noteworthy advances and discoveries in each department published at the head of each of the several topics or branches dealt with. This enables the reader to grasp the novelties and improvements in a few moments, hence, it cannot fail to be an acceptable addition. Illustration has been used with considerable liberality, there being fourteen full-page plates, besides numerous pictures throughout the text, especially in the surgical volume. The Gould-Saunders' yearbook continues to maintain the supremacy established for itself in the beginning by editor and publishers.

A Reference Handbook of the Medical Sciences. Embracing the Entire Range of Scientific and Practical Medicine and Allied Science. By Various Writers. A new edition, completely revised and rewritten. Edited by Albert H. Buck, M. D., New York City. Nine volumes, imperial octavo. Volume VII. Illustrated by chromolithographs and 688 half-tone and wood engravings. New York: William Wood & Co. 1902. (Price, muslin, $6.00 per volume; leather, $7.00 per volume; half morocco, $8.00 per volume.)

This is the day of epitomes, condensations, yearbooks, and dictionaries of medical science. A busy doctor wants information on some urgent points and he wants it quickly,—now. As a matter of fact, however, he needs something more than a mere hint; he requires a condensed history or abstract of the subject in hand, and in this reference handbook, *par excellence,* he gets just what he most needs,—namely, a succinct, carefully prepared synopsis of every topic of practical value in the whole domain of medical science.

This seventh volume embraces topics from Sacc to Ulc of the alphabet, and contains some of the most important material in medicine; for example, diseases of the spine and spinal cord, syphilis, tuberculosis, and typhoid fever. The articles on the spine and spinal cord are almost exhaustive, so thorough are they in description and treatment. The same might be said with a truth of the other three titles named, as well as of still others in the book not mentioned. Two more volumes will finish the series and it then will form the most complete reference handbook that has been published. No reference library can be considered as properly equipped without a set of these books.

---

Clinical Pathology of the Blood. A Treatise on the General Principles and Applications of Hematology. By James Ewing, M. D., Professor of Pathology, Cornell University Medical College, New York. Second edition, revised and enlarged. Octavo, 492 pages, with 43 engravings and 18 full page colored plates. Lea Brothers & Co., Philadelphia and New York. 1903. (Cloth, $3.50, net.)

The blood is receiving but recently the study to which its importance, both physiologically and pathologically, entitles it; or, to state the point more directly, pathologists are beginning now to investigate this important fountain-head of many diseases which long ago wrought havoc with human life. That the blood stream as a disease carrier should have escaped observation so long is almost incomprehensible. However, we are now in the flood-tide of this neglected domain and our investigators are making surprising progress in this field of discovery. Ewing, the author of the book before us, ranks as one of the foremost students in this branch of science and has given the profession one of the best books published in relation to the pathology of the blood. The first edition of two years ago became exhausted early and is replaced by this one which has been improved by the inclusion of whatever of value has been brought out since the appearance of the original book.

Only a very meager conception of the great labor involved in a revision of this treatise may be formed from the statement that 400 new articles have been examined and added to the bibliographical lists and their principle points incorporated in the text; that changes have been made relating to technic, the serum test for blood and crioscopy; also enlargement of the presentation of morphology of the blood; additions of the topic of leukemia; presentation of Ehrlich's theories on immunity, and several other improvements in text and illustration, which serve to make this a conspicuous and reliable textbook on the blood.

A Manual of Bacteriology. By Herbert U. Williams, M. D., Professor of Pathology and Bacteriology, Medical Department, University of Buffalo. Duodecimo, pp. 351. With 99 illustrations. Third edition, revised and enlarged. Philadelphia: P. Blakiston's Son & Co. 1903. (Price, $1.75.)

A good book treating on an important subject is sure to pass through a number of editions. The work before us is getting its just deserts in the direction indicated. We have expressed heretofore, in noticing previous editions, our opinon of this manual,—an approval that we maintain unchanged as we examine this latest issue.

The text has been prepared with special reference to the needs of a beginner in the study of bacteriology and, also, to meet the clinical requirements of the physician. Such additions as have been made, to keep it abreast of the present time in its scientific aspect, have not materially increased the size of the book. New illustrations have replaced some of those in former editions, and some other changes have been made which give freshness to its appearance and bespeak the spirit of progress which characterises the work of its distinguished author. The chapter on disinfectants and antiseptics, by Dr. Thomas B. Carpenter, and one on the Surgical technic of disinfection, asepsis, and antisepsis, by Dr. Marshall Clinton, are of great practical value, and deserve the careful study of the beginner in surgical work, but especially of the careless surgeon. As a whole, the Williams manual has not been excelled by any similar work in the book market.

---

International Clinics. A Quarterly of Illustrated Clinical Lectures and especially prepared original articles on Treatment, Medicine, Surgery, Neurology, Pediatrics, Obstetrics, Gynecology, Orthopedics, Pathology, Dermatology, Ophthalmology, Otology, Rhinology, Laryngology, Hygiene and other topics of interest to students and practitioners. By leading members of the medical profession throughout the world. Edited by A. O. J. Kelly, A. M., M. D., Philadelphia. Volume IV. Thirteenth series. 1904. Philadelphia: J. B. Lippincott Company. 1903. (Cloth, $2.00.)

This volume includes articles on treatment, medicine, surgery, gynecology and obstetrics, neurology, orthopedics, ophthalmology, and pathology. Among the foreign contributors are the names of Albanan, Battle, Burnet, Corner, Duckworth, Duncan, James, Julien, Pinard, Poynton, and Valude. The home lecturers include the names of Brower, Clayton, Coomes, DaCosta, Davenport, Dugan, Favill, Frank (Louis), Keen, McFarland, Musser, Porter, Preble, Satterthwaite, Senn, Tyson, Wiggin, and Wood.

The subjects of these lecturers cover a wide range of topics and the material cannot fail to interest specialists, as well as general practitioners. The illustrations are of excellent quality, though not as numerous as could be wished. Dr. Davenport's article on displacements of the uterus is the best illustrated of any in this volume and will prove of great value to the country practitioner. The high standard of these clinics continues and with steady improvement.

Clinical Treatise on the Pathology and Therapy of Disorders of Metabolism and Nutrition. By Prof. Dr. Carl von Noorden, Physician-in-Chief to the City Hospital, Frankfurt a. M. Authorised American edition, translated under the direction of Boardman Reed, M. D., Professor of Diseases of the Gastrointestinal Tract, Hygiene and Climatology, Department of Medicine, Temple College; Physician to the Samaritan Hospital, Philadelphia. Part IV. The Acid Autointoxications. By Prof. Dr. Carl von Noorden and Dr. Mohr. Duodecimo, pp. 80. New York: E. B. Treat & Co. 1903. (Price, 50 cents.)

A monograph on this subject forms useful reading for a busy physician. The various phases of the subject are considered under the following heads: General remarks on autointoxication with acid products of metabolism; the sources of the acetone bodies; where are the acetone bodies formed? pathological nondiabetic acetonurias; diabetic acidosis; and therapeutic considerations.

It is a book that can be carried in the pocket, hence may be used to refresh one's thoughts during travel. The scientific treatment of the topics dealt with is most gratifying.

